# Coatings as the useful drug delivery system for the prevention of implant-related infections

**DOI:** 10.1186/s13018-018-0930-y

**Published:** 2018-09-03

**Authors:** Chenhao Pan, Zubin Zhou, Xiaowei Yu

**Affiliations:** 1 Department of Orthopaedic Surgery, Shanghai Jiao Tong University Affiliated Sixth People’s Hospital, Shanghai, 200233 China; 2 Department of Orthopaedic Surgery, Shanghai Sixth People’s Hospital East Campus, Shanghai University of Medicine and Health Sciences, Shanghai, 201306 China

**Keywords:** Implant-related infections, Coating, Osteomyelitis, Local drug delivery system, Biofilm

## Abstract

Implant-related infections (IRIs) which led to a large amount of medical expenditure were caused by bacteria and fungi that involve the implants in the operation or in ward. Traditional treatments of IRIs were comprised of repeated radical debridement, replacement of internal fixators, and intravenous antibiotics. It needed a long time and numbers of surgeries to cure, which meant a catastrophe to patients. So how to prevent it was more important than to cure it. As an excellent local release system, coating is a good idea by its local drug infusion and barrier effect on resisting biofilms which were the main cause of IRIs. So in this review, materials used for coatings and evidences of prevention were elaborated.

## Background

Implant-related infections (IRIs) are the result of bacteria adhesion to an implant surface and subsequent biofilm formation at the implantation site [1]. The incidence of IRIs in orthopedic trauma patients was from 5 to 10% depending on the severity of the injury, condition of soft tissue, and the type of fracture [2]. It remains challenging and expensive to treat IRIs, despite advances in antibiotics and new operative techniques. The traditional management of IRIs includes irrigation and debridement, obliteration of dead space, intravenous antibiotics, and removal of the hardware [3]. Each year, 750,000–1,000,000 IRIs occur in the USA, and the government needs to spend more than $1.6 billion to cover the expense of the excess hospital charges [4]. Especially, with the widely use of orthopedic implants, the number of infected implants was continued to increase [5]. Even if the infected implants can be successfully removed by secondary surgery, the functionality of the limb and the fracture healing may be limited, which may eventually lead to fatal surgical operations such as amputation, joint arthroplasty, or arthrodesis.

So how to prevent the occurrence of fatal IRIs is more important than the treatment. IRIs are typically caused by microorganisms which grow in biofilms and adhere to the implant surface in a highly hydrated extracellular matrix. Avoiding biofilms forming can effectively prevent or treat IRIs [6–9]. As the elective surgery could not be performed under an absolutely sterile environment, bacteria may prefer to adhere to the surface of the bioinert titanium implants and form biofilms, especially when the host’s immunological defense functions are compromised and/or the systemic antibiotic prophylaxis is not very effective [10, 11]. Bacteria which can resist immune responses in biofilms were much less susceptible to antibiotics [12]. Therefore, it is difficult to truly eliminate the biofilm infections and, typically, there are chronic recurring symptoms, even after antibiotic therapy. So the prevention of the growth of nosocomial pathogens is more important than the elimination of the biofilm in IRIs.

Because of the restrictions of traditional systemic drug treatment of bone infection, such as poor effect or hepatorenal toxicity, drugs for IRIs should be performed locally and specifically for implants sites at optimal concentrations over appropriate stages [13].

Numerous strategies have been attempted to prevent and treat IRIs by either implant surface fabrication or incorporation of antibiotics into the implant devices. Recent developments in material science showed that implants with biodegradable polymer coatings can be used as controllable means to deliver antibiotics in a sustained fashion. Polymer coatings are capable of completely releasing all antibiotics in a sustained fashion thus minimizing any local or systemic toxicity associated with high fluctuating antibiotic concentrations. For example, Buchholz et al. [14] reported that implant with a synthetic polymers coating as a local drug delivery system significantly reduced the infections and representing a promising approach in the treatment of IRIs. One of the main advantages of implant surface coating-mediated local drug delivery is keeping other parts of the body out of affected so as to avoid serious systemic side effects [15]. A sustained and high antibiotic concentration over minimal inhibitory concentration (MIC) of pathogenic bacteria at the implant site is expected to inhibit bacterial adhesion, colonization, and biofilm formation [16].

The antibacterial implant coating can be divided into calcium or silicon bone cements, polymer hydrogels, and antibacterial ion coatings based on the materials selected by different manufacturing processes like spraying, smearing, and electroplating [17, 18]. In this review, we will focus on (1) implants coated with antimicrobial substances and (2) the usage of coating in prevention of IRIs.

## Mechanism of the IRIs—biofilm

Biofilms are aggregates of microorganisms as a self-produced matrix of extracellular polymeric substances (EPS) where bacteria are frequently embedded. EPS adherent to the medically surface (skin, implants, wearing) are accounting for most of microbial infections in the internal fixation devices [16]. Bioinert surfaces attract the biofilm formation [18–20]. The ligand of bacterial fimbriae can be bound to electrovalent bond or hydrophobic bond on the surface of the material. The factors of a surface that determine initial bacterial attachment are its hydrophobicity and roughness [19–21].

In most previous studies on bacterial adhesion on titanium and ceramic surfaces, the quantity of bacterial adhesion showed a direct positive correlation with surface roughness [22–24]. From an atomic force microscopy (AFM) viewpoint, most surfaces are rough and all kinds of surfaces provide adequate conditions for bacterial adhesion [25]. According to the thermodynamic model of microbial adhesion, hydrophobic materials are preferentially colonized by hydrophobic bacteria [26–28]. Consequently, the adhesion properties of different bacteria are affected by the hydrophobicity of the bacterial cell surface [29, 30]. Both *S. aureus* and Methicillin-resistant *Staphylococcus aureus* (MRSA) which are common bacteria in IRIs are known to prefer hydrophobic surfaces [31, 32]. Titanium implants are often bioinert, smooth (Ra = 280 nm) but enough for bacterial adhesion [33] and hydrophobicity to prevent blood clotting. Thus, biofilm formation occurs commonly [22–33].

Biofilms are complicated systems with high microorganisms’ densities, ranging from 10^8^ to 10^11^ bacteria g^− 1^ wet weight [12]. Most of the biofilm biomass comprises hydrated EPS instead of bacteria. The intermolecular interactions among EPS components originating from self-organization of EPS matrix determine the mechanical characteristics and the biological activity of the matrix in the biofilm [12]. The biofilm architecture formation is a continuous process that creates a micro spatial organization where bacteria clusters present in the biofilm in micro colonies. As the ramparts of bacteria, biofilms have the feature of antibiotics tolerance.

Antibiotics tolerance of biofilms is due to the properties of the biofilm matrix and of the slow growth which occurs in biofilms. The components of EPS matrix can deactivate antimicrobial substances diffused through the biofilm as diffusion–reaction inhibition [34, 35]. Antimicrobial resistance may be promoted by diffusion–reaction inhibition form biofilms through decreasing the effective concentration of antimicrobials that bacteria are exposed to. On the other side, dormancy and slow growth rates have been considered to be ways of bacteria survival in biofilms being exposed to antimicrobials for a long time [36]. The formation and antibiotics resistance of biofilm is time-dependent. The initial bacterial attachment (within an hour) is crucial for the biofilm formation [37–39]. Cell wall-anchored (CWA) proteins of bacteria promote attachment to surfaces in the following 24 h bacteria adhesion stage. The scanning electron microscope (SEM) revealed implants surface was comprised of bacteria clusters always associated with fibrils, which was presumed as fibrin, and surrounded by diameter host cells [35, 37]. When the bacteria are anchored to the implants, biofilm formation begins to develop. There was a new structure which was called “lacunae” till day 7 [15, 35]. The lacunae is the shallow depressions consistent with the size of bacteria, which meant the matrix spaces left by dispersed bacteria. The accumulation of bacteria during biofilm formation is attributed only to the polysaccharide intercellular adhesin (PIA) [38–40]. Next, the biofilm proliferation and maturation remodeling by phenol-soluble modulins (PSMs) begin [41]. After 14–28 days, empty lacunae and a few bacteria were the main morphological characteristics of EPS matrix. This phenotype of EPS matrix remained the same till 6 months post-implantation and showed an unexpectedly outstanding stability of EPS mature biofilm in chronic implant-related infection [35]. In summary, typical biofilm formation is first shaping at day 7 and its growth diffusion peaks covering 30–40% of the implant is at 2 weeks.

Unlike the distinct biofilm formation phenotypes in vitro, the in vivo biofilm formation comprised of strains is hard to be indistinguishable [42]. The biofilm formation in vivo takes longer time than in vitro, which may be due to the “race to surface” between bacteria and host cells. Bacterial attachment and biofilm formation stage lasted 12–24 h, and the biofilm proliferation and maturation lasted 36–72 h for completion [43, 44]. The early infection may be defined up to 3–4 weeks during which debridement and antibiotic therapy with the retained stable implants were performed in the traditional management perspective [45, 46]. The residual biofilms on retained implants may cause the recurrence of IRIs in many clinical cases. Bioinert polymer coatings like polymethylmethacrylate (PMMA) [47] with antibacterial agents have been used to prevent early fibrin and bacteria adhesion through its barrier and antibacterial effect for IRIs [48, 49].

Over time, the complete biofilms are gradually formed, so is the antibiotic resistance. On the basis of time- and dose- dependent effect of antibiotic susceptibility [50–53], ideal cumulative prevention and cure antibiotics release kinetics of the coatings should have the releasing peak over the minimal bactericidal concentration (MBC) during 7–14 days that prevent biofilm formation followed by sustained release between the MIC and MBC over several weeks. By inhibiting the biofilm formation, cells are in a dominant position in the competition against bacteria for growth, so the ideal releasing should have the concentration above MIC over at least 28 days.

At least 1% bacteria in stationary phase in biofilms are tolerant to antibiotics [54]. As time goes on, more bacteria in the biofilm moved into the stationary phase. Hence, for some kinds of antibiotics like vancomycin, antibiotics tolerance of biofilms showed temporal correlation, which denoted that higher tolerance was shown in older biofilms for these antibiotics as well as metal nanoparticles like Ag [34]. Moreover, biofilms would always die from the outside-in instead of the inside-out [53]. According to the characteristics of biofilm, the treatment is more difficult than prevention. Depending on the type of fracture and contamination of the trauma, second operation for IRIs may be avoided by the use of coating with the ideal antibiotics release curve for prevention.

## Main components of coating for IRIs

### Bone cement coatings

Various forms of PMMA bone cements and the beads made of it with antibiotics have been used for more than 40 years in hip replacement or in acute and chronic osteomyelitis [45, 47, 55, 56]. There are several premixed cements that have been allowed the clinical use by American Food and Drug Administration (FDA): DePuy® (DePuy Orthpedics), Cobalt™ G-HV (Biomet), Cemex® Genta (Exactech), Palacos® G (Biomet), and Simplex® P (Stryker Orthpedics). However, these FDA approved antibiotics eluting PMMA bone cements are more appropriate being used as a preventative measure than for the treatment because of their lack of effect on active IRIs due to limited and burst release of embedded antibiotics [57, 58]. Antibiotics release from PMMA cements is mainly due to the diffusion through surface roughness, superficial pores, and surface erosion [46, 58, 59]. The release characteristic of PMMA was typically performed as a biphasic phase which included a burst head of initial release and a continued tail of ineffective release for weeks or months. The mechanical strength of PMMA is satisfactory, but the low efficiency of the local drug release is the main barriers for its clinical use in IRIs. Therefore, a variety of new biomaterials have been developed and manufactured as alternative antibiotic-eluting bone cements.

Calcium phosphate cements (CPC), beta-tricalciumphosphate (β-TCP) and hydroxyapatite (HA) are the calcium phosphate materials. The calcium phosphate materials are degradable and the end products are calcium and phosphate (Ca^2+^, PO_4_^3−^) that are biocompatible, bioactive, and stimulate new bone growth [59–62]. The injectable CPCs are now commercial available that can be solidified after implantation. [60, 61]. There are commonly two phases before the CPCs solidified: the particles and the liquid for better performance. CPCs have the ability of self-set, self-molding, and no exothermic reaction which may be harmful for the bone and incorporated drug [59, 62]. The slow biodegradation in vivo and poor biomechanical strength have restricted the clinical application of CPCs [58]. In addition, the microstructures of CPCs are dense, lacking of macroporosity that are obviously not suitable for the adhesion, penetration, and colonization of cells and tissue regeneration [58].

Antibiotics, such as gentamicin [46, 63–66] and vancomycin [67, 68], can be mixed in the liquid phase of calcium phosphate cement, HA, or β-TCP [46] against *S. aureus* and MRSA. The apatite cements are made up of microcrystals that have a better biological performance in size and formation than HA particles [63], and show better antibacterial activity. HA nanoparticles can be propagated by wet chemical precipitation and show good bactericidal effect for implant-related pathogens through toxic effect of damaging bacterial membrane [69–71]. It was reported that a drug-chitosan compound was filled in the porous HA matrix and subsequently coated onto the implant smooth surface to obtain a Ti_6_Al_4_V implant with drug-chitosan-HA-coating [72–74]. The burst releasing peak continued in the first several hours, and the continued release were covered the post-operative time of perioperative period or 4–8 days. The rest releasing was lasting for more than 1 month. Though HA have better biomechanical properties, β-TCP seemed to be more suitable for drug release. Another coatings comprised of HA and TCP were defined as bi-phasic calcium phosphates (BCP). More ions dissolved in the BCP in the local releasing, which meant more carbonate hydroxyapatite on the surface [75]. A β-TCP coating contained doxycycline (BonyPid™) was described to form a steady, zero-order rate releasing up to 30 days to be capable of eliminating the contaminating bacteria [70]. Moreover, poly(lactic acid)(PLLA)/β-TCP coating presents a good result of infection as manifested by the microbiological, radiological, and histological analysis [67, 76]. At present, the releasing profiles of FDA-approved antibiotic eluting bone cements have the limitations of burst and limited release that are not sufficient to reach a desired constant and long-term sustained release effect to satisfy time- and dose-dependence of biofilms prevention.

### Hydrogels coatings

Hydrogels are usually prepared from natural to synthetic polymers with high degree of hydration and represent promising biomaterials for tissue engineering and regenerative medicine. One class of natural polymers is polysaccharides (e.g., dextran, alginate, chitosan, fibrin, and proteins gelatin). Synthetic polymers include polyethylene oxide (PEO)/polyethylene glycol (PEG), poly (vinyl alcohol) (PVA), poly (acrylic acid) (PAA), and others [49]. Implant surface with PEG and/or PEO coating can endow the ability of anti-bacteria adhesion [77, 78]. There was a report that the inclusion of arginine-glycine-aspartic acid (RGD) array restored the function of local osteoblasts that were damaged by some synthetic polymer coatings [79]. A solubilizing surrounding was offered for the antimicrobials dissolution in local delivery system by the hydrophilic characteristic of hydrogels. Several antibiotics, like ciprofloxacin [80], amoxicillin [81], and gentamicin [82] can be embedded in the bioactive hydrogels. Hydrogels were recently designed by self-assembly formation of tripeptide (D)Leu-Phe-Phe with the incorporation of ciprofloxacin [49, 80]. During the self-assembly process, it showed that the antimicrobials played an active role in process to incorporate into the hydrogels formation directly. The non-covalent bond contributed to the antimicrobials integration in peptide structure [48, 49]. The final release was indicated to reach the effect of anti-infection.

The antibacterial properties of chitosan is due to the combination between positively charged amino groups of chitosan and negatively charged bacterial membranes, leading to bacteria membrane leakage [83]. However, because of the displayed weak positive charges on chitosan, coating with chitosan reveal only limited antibacterial effects [84]. Furthermore, the physical and chemical characteristics of chitosan are poorly for embrittlement at room temperature and in acid dissolution environment [85]. Another hydrogel similar to chitosan is a polysaccharide originated from natural chitin polymerization and has the antibacterial activity [86]. The bacterial adhesion and subsequent biofilm formation can be prevented by using chitosan alone. Several temperature-responsive structures of hydrogels (PLLA-PEG-PLLA, PDLA-CPC-PDLA, and PDLA-PEG-PDLA) were reported [87]. These hydrogels were performed to inhibit the growth of *S. aureus* and *E. coli*.

Titanium implants with different antibiotics (like gentamicin)-doped hydrogels coating significantly prevented the occurrence of infection. However, the methods of antibiotics hydrogel coating need to be optimized to reach suitable drug releasing profiles and optimal coating matrix degradation rate. Controlled delivery system loaded with drug on the titanium implants showed the double ability of antibacterial and osteogenesis forming by biodegradable sol-gel and polymers coating [88, 89]. The controlled releasing technology of nanostructured sol-gel provided a continuous and effective local delivery system for orthopedic instruments to prevent and cure IRIs [90].

### Silver/silver ions coatings

Most of the metal ions coating is in the form of ionic or nanoparticles instead of bulk material [91]. Silver ion is bioactive by wrecking the cell membrane permeability and cellular metabolism [91, 92]. The sulfhydryl groups of metabolic enzymes and bacterial DNA are disrupted by Ag to the destruction of bacterial membranes, so the bacterial key metabolism and replication are inactivated [92]. Implants coated with silver can prevent bacteria adhesion and subsequent colonization (like *S. aureus* and *S. epidermidis*) in vitro [93, 94]. Titanium dioxide itself has been performed to be a kind of anti-infective biomaterials or cooperated with other factors [95, 96]. However, implant with low-dose silver coating (< 1 ppm) may be incapable of reducing the infection rates [97], while high-dose silver coating (> 1.5 ppm) may induce cytotoxicity [98]. However, cytotoxicity sensitivity is different among different cells [99–101]. Therefore, for the simple silver coating, the concentration of silver had better range from 1.8% to 6.5 wt% for inhibiting bacteria proliferation without decreasing osteoblast activity [99, 102]. No observable adverse effect level (NOAEL) of silver is up to 30 mg/kg [103]. The most serious adverse effect of silver is Argyria, which is not found at or below 1.7 g total silver in vivo. For a long time release, the minimum requirement for antibacterial effect was at the concentrations of at least 0.1 ppb [104, 105]. Another study reported that an optimal silver density on the implant surface was 1 × 10^18^ ions/cm^2^, representing a balance between corrosion resistance and antibacterial effect [106]. Unlike Ag^+^ which is diffused to the surrounding tissue, a new antibacterial HA film was developed by immobilization of Ag in HA film through inositol hexaphosphate chelation. This antibacterial HA film coating demonstrated excellent antibacterial activity both in vitro and in vivo [107]. Data generated from a *S. aureus* induced in vivo osteomyelitis model demonstrated that no bacteria infection was detectable up to 21 days after implantation of implants with antibacterial HA film coating [107]. Ag-coated fracture external fixation pins were examined in human studies; however, these studies fail to demonstrate any advantages in reduction of pin site infections when silver-coated pins were used [108].

In summary, most studies have concentrated on these three kinds of materials coating. Other coating materials, such as iodine ions [109, 110], titanium oxide photocatalysis, nitric oxide, and graphene coating [111], have provided new ideas and alternative approaches for the prevention of IRIs.

## Evidence of the anti-infective prophylactic therapy of coating

### In vitro studies

Many studies using antimicrobial coatings have achieved on IRIs prevention in vitro. The therapeutic effects of antibacterial coating materials on the prevention of biofilm are usually evaluated by the in vitro zone inhibition of bacterial growth, such as *S.aureus* or MRSA [69, 112], antibiotic release profiles and bioactivities [70, 113, 114], cytotoxicity [67] of osteoblast, or other cell lines from the liver and kidney [104]. A characteristic time- and dose-dependent sustained antibiotics release is critical for the biofilm formation inhibition. David et al. tested a variety of antibiotics releasing efficacy and showed that all antibiotics used alone or in combination showed an initial burst release peak with dose-dependent antimicrobial effects and have no negative effects on osteoblast [112]. Local antibacterial spectrum and releasing curve for the biomaterial were needed to be considered when coating with antibiotics. The synthesized HA nanocrystals displayed antimicrobial effect against the IRIs by damaging bacteria membrane [69]. HA nanoparticles dispersed in the chitosan matrix lowered the burst releasing peak of the small molecule drug because of HA physisorption, which promoted persistent release kinetics up to 3 weeks [70]. The calcium phosphate coating can reduce burst release, which provided long-term release kinetics throughout 4 weeks [113]. Another team coated vancomycin onto PLLA/β-TCP composites to release antibiotics through dip coating. The PLLA/β-TCP coating was biocompatible on cell proliferation, adhesion, and mineralization [67]. While gentamicin-doped poly(D,L-lactic acid) (PDLLA) coatings had an initial burst release and around 60% of incorporated drug was released within 1 min. Then, a subsequent slow and constant release of gentamycin was observed lasting 6 weeks from 40% down to 15% [114].

Implants with antibiotic-doped coating suffered from a problem that antibiotic therapeutic concentrations lasted for a limited period time. When the antibiotic was exhausted, the antibiotic concentration was down to the levels enabling bacteria to escape antibiotic and colonize the implants [115]. Alternative approaches have been developed to tethering antibiotics on the surface of metal implants through a bridge linker and forming an immobilized antibacterial coating. The immobilized antibacterial coating is expected to be functional throughout the whole life of implants. Various types of antibiotics and bacterial membrane destructing molecules are suitable candidates for the preparation of immobilized antibacterial coating [116]. Compared to antibiotics, anti-microbial peptide was highly specific and had minimal toxicity to cells without drug resistance [117]. However, anti-microbial peptides are preferred for the treatment of IRIs instead of prevention.

### In vivo studies

For mouse and rat model, 10^5^ colony-forming unit (CFU) bacterial suspension is often injected into the tibia bone marrow and fixed with different material steel pins [64, 118–120] Rat tibia implantation with bacterial infection[121] can be initiated by inoculation of a bacterial suspension at the site of implantation [122] or the use of a pre-colonized implant1. Mixed-models, involving the use of both a pre-colonized implant and a bacterial suspension [123], were reported to reproduce the clinical use of an infected implant and contaminated washing solutions at surgery, respectively. One limitation of the mixed models was the difficulty in distinguishing the biofilm formed on the implant from that of the inoculated bacterial suspension. Previous studies showed that inoculation of between 10^2^ and 10^6^ CFU of *S. aureus* showed similar histological changes [124, 125]. Results from in vivo were similar to in vitro demonstrating that biofilm was found on the implant surface on day 1 and then robustly proliferate on day 3, persisting until day 7. Biofilm formation was steady at 40% covering on day 14 [35]. Layer-by-layer (LBL) coatings were designed using the electrostatic multilayer assembly to get a programmable release [49], which was able to allow release antibiotics contained in upper layers in early stage that prevented the formation of biofilm followed by sustained antibiotics release in lower layers above MIC for 3–4 weeks [118]. These kinds of coatings provide two kinds of releasing curve to overcome drug deficiency during slow releasing. Beside antibiotics coatings, lysostaphin has been coated on the titanium implants and tested in a mouse model. Bacterial growth was almost totally inhibited, and a successful osseous healing was observed with lysostaphin-coated implants [119]. As mentioned earlier, ionic silver was immobilized on implant with hexaphosphate chelation by low heat immersion process. In mouse osteomyelitis model, there was no detectable bacterial presence 3 weeks after inoculation with *S. aureus* without burst releasing [107]. Gentamicin coating on titanium implants with gentamicin-sodiumdodecylsulfate (SDS) and tannic acid demonstrated a high preventive effect on IRIs, which showed successful rapid osseointegration [64]. The rapid osseointegration is necessary which meant repairing completed. Gentamicin palmitate coating also significantly reduced IRIs on the implants as well as systemic inflammation [120]. PLLA/β-TCP-coated implants loading with vancomycin presented favorable controlling IRIs and advancing bone osseointegration for 6 week [68].

Rabbit osteomyelitis model has been widely used for the evaluation of the antibacterial effect. Before fixed with different material steel pins, 10^6^–10^8^ CFU bacterial suspension is injected into the tibia bone marrow [126–128]. Polymer-Lipid Encapsulation MatriX (PLEX)-doxycycline-coated implants successfully avoided the occurrence of IRIs, even when rabbits were inoculation with a doxy-resistance strain [126]. The chitosan-calcium sulfate coating was reported to improve the therapeutic and preventive outcome of IRIs by prolonging the period of high releasing concentrations of antibiotic [127]. Ti_6_Al_4_V pin coated vancomycin–chitosan/HA [74] and phosphatidylcholine [128] reached the similar results for the prophylaxis and therapy of IRIs. Local antibiotics release system can be easily used in surgery for IRIs by inhibiting biofilm biofilms formation on the implant surface [74, 128]. The abovementioned coating strategies are based on the traditional user-friendly technology and have some limitations in the release curve. While LBL formation by the deposition of hydrolytically poly (b-amino ester), poly (acrylic acid), and gentamicin were constructed without pre-modification. Interestingly, a burst-release peak of drug was over several days and followed by stable continuous zero-order release for weeks by hydrolytic erosion [63].

There are relatively few studies reported in using large size (dogs, miniature pigs, goat) animal models. Tran et al. created a titanium oxide combined with siloxane polymer coating doped with silver on intramedullary nails by metal-organic methods and tested in a goat osteomyelitis model with fracture. The tibia shaft fractures were created followed by the injection of 2 × 10^4^ CFU bacterial suspension in the osteotomy site. Then, a silver coated intramedullary nail was inserted for fixation. 5 weeks after fixation, the cured goat can walk by all four limbs without infection compared to the unwilling walking control, which suggested that the coated intramedullary implant as local antimicrobial releasing system for IRIs was feasible and effective [102]. In this research, the injection of bacterial suspension is low. Salgado CJ et al. established a model of tibial osteomyelitis by the injection of 4 × 10^9^ CFU bacterial suspension into osteotomy site [129]. However, there is lack of evidence to make sure the concentration of bacterial suspension for large size animal models.

### Clinical studies

Until now, large randomized and multicenter clinical studies using antibacterial coating for the prevention of IRI were hard to be executed, because of the diversity of anti-infective coatings and the specificity of the manufacturing processes. Expert Tibia Nail (ETN) PROtect™ coated with a biodegradable gentamicin-laden polymer was used for IRIs. When it was used for the prophylaxis of osteomyelitis, no deep infections were observed after the placement of the gentamicin-coated nail and no side effects were reported that were linked to the implant coating [65, 130]. Conway et al. used antibiotic cement-coated (ACC) rods for the control of IRIs by providing both the mechanical stability and local delivery of antibiotics. In this research, 60% of the patients were cured after the first procedure using ACC for infected arthrodesis and infected non-union and gets better functional rehabilitation. At last follow-up, 5 patients need amputation, illustrating a limb salvage rate of 105/110 (95%) [131]. In the operating room Thonse et al. has used interlocking intramedullary nails coated with antibiotic cements before implantation. They found that the infection was well controlled in 95%(19/20) of treated patients, while the rest 1 patient had a union with infection and underwent an amputation unfortunately [132]. In a prospective, non-randomized case series, Fuchs et al. investigated the outcomes of 21 patients underwent surgical treatment with the gentamicin-loaded coating of an implant (UTN PROtect), and no implant-related infections occurred [133]. A 17-year-old man with grade IIIc tibial fractures was also treated by UTN-PROtect and has been successfully limb salvaged instead of prolonged external fixation or amputation which were the standard treatment [66]. Silver coated on the implant surface (Mutars® tumor endoprosthesis (Implantcast, Buxtehude, Germany)) reduced the infection rate from 17.6 to 5.9% in patients with bone sarcoma [134]. Knee arthrodesis nail based on Mutars® technology was successful in eradicating infection [135]. A case control study was conducted to examine the effect of silver-coated coatings, which showed the post-operative infection rate was 11.8% compared with 22.4% for the control group [136]. However, a report showed that local argyria occurred by Mutars® tumor endoprosthesis though the length of the implant did not influence the development of local argyria [137]. Implants coated with iodine can be effective for preventing and treating IRIs. Two case series indicated that the rate of preventing or treating IRIs was over 95% with cytotoxicity and adverse effects [109, 110]. A summary of above mentioned clinical studies was listed in Table 1. The evidence of the research was weak because of the number and design of cases although kinds of coatings showed excellent effective for preventing and treating IRIs.

**Table 1:** Published clinical data of different coatings

Authors	Implant	Coating technology	Study type	Evidence level
Moghaddam et al. [65]	Tibia nail (ETN PROtect™)	Gentamicin PLLA with “dip coating process”	Case series	IV
Metsemakers et al. [130]	Tibia nail (ETN PROtect™)	Gentamicin PLLA with “dip coating process”	Case series	IV
Conway et al. [131]	Rods (antibiotic cement-coated)	Tobramycin and vancomycin cement (Biomet Cobalt) with metal molds or silicone tubing	Case series	IV
Thonse et al. [132]	Interlocking intramedullary (antibiotic cement-coated)	Antibiotic powder Interlocking intramedullary wrap-ped by cement (Zimmer, Warsaw, Indiana) with metal molds	Case series	IV
Fuchs et al. [133]	Tibia nail (UTN PROtect)	Gentamicin PLLA with dip coating process	Case series	IV
Raschke et al. [66]	Tibia nail (UTN PROtect)	Gentamicin PLLA with “dip coating process”	Case report	IV
Hardes et al. [134]	Endoprosthesis (Mutars)	Silver with galvanic deposition	Case control study	III
Wilding et al. [135]	Knee arthrodesis nail (Mutars)	Silver with galvanic deposition	Case series	IV
Wafa et al. [136]	Silver-enhanced custom-made endoprostheses	Silver with anodisation of the titanium alloy	Case control study	III
Glehr et al. [137]	Endoprosthesis (Mutars)	Silver with galvanic deposition	Case series	IV
Shirai et al. [109]	Kyocera Limb Salvage System KOBELCO K-MAX	Povidone-iodine electrolyte-based process	Case series	IV
Tsuchiya et al. [110]	Spinal instrumentation, plates, external fixator pins, prostheses, nails, cannulated screw	Povidone-iodine electrolyte-based process	Case series	IV

## Conclusion

Prevention is better and more important than treatment for IRIs. The characteristics of time- and dose-dependence of biofilm formation require a more constant and sustained antibiotics release from implant coatings. Surface coating as one of implant surface fabrication approaches has been extensively investigated for the purpose of preventing and treating IRIs by either local antibiotics eluting or forming an antibacterial surface to resist the biofilm formation. A desired antibacterial implant coating is expected to enhance the adhesion and growth of host cells, while inhibiting bacterial adhesion and biofilm formation, so that host cells can be the winner in the “race to surface.” The formation of biofilm and antibiotic resistance are time- and dose-dependent, so that the antibacterial effect of coating should be sustained and constant at effective concentration at least for 4 weeks with a short-term releasing peak at 4–7 days. According to the characteristic of biofilms, layer by layer coating was more appropriate than monolayer coating for IRIs prevention.

*S. aureus* is one of the leading pathogens involved in IRIs [138]. As shown in Tande et al.’s [139] study that summarized the microbiological results of 2400 patients with IRIs, around 60% of IRIs was caused by *S. aureus*. Patients with *S. aureus* IRIs frequently have multiple medical comorbidities [140], such as diabetes (30 to 40%) [141] and rheumatoid arthritis (10 to 20%) [142]. Gentamicin and vancomycin are commonly used antibiotics for local drug eluting. For many years, antibiotic (gentamicin and vancomycin)-impregnated PMMA bone cement has been widely used to prevent IRIs [47]. Though PMMA cement is mechanically strong, the therapeutic efficacy of this treatment was questioned in recent studies. Major problems with antibiotic-loaded cements are their burst release and limited release of embedded antibiotics because of the diffusion through surface roughness, superficial pores, and surface erosion [46, 59]. It is estimated that over 90% of loaded antibiotics are retained within the PMMA cement [58]. Regarding the IRIs prevention, the polymers hydrogel coating significantly extended the antibiotics release. The hydrogels release the drug steadily through the crosslinking structure [78–81]. Drug release is closely related to gel degradation through chemical bonds or ionic bonds [82–85]. The silver or silver ion coating seems to be the best way of prevention because of its broad antibacterial spectrum, a stable time- and dose-releasing effect, stable structure. But it has certain side effects on the human body, even if it is partially sustained releasing.

There are great opportunities and challenges to construct an ideal local drug delivery system. It is necessary to further establish a polymer system that has appropriate mechanical strength, matrix formation, desired drug-releasing profiles, and is biodegradable in clinical application. Through data generated from many in vivo and in vitro studies were promising, there was lack of clinical trials for further validation. Therefore, future research should be concentrated on the clinical evaluation of polymer systems such as their clinical efficiency and analysis of post-operative surface of coatings. As for functional groups, drugs and agents can be improved in embedding method to achieve an ideal releasing curve, active ingredient, and conventional drug in new use or effective factors loaded.

In short, for modified implants, translational medicine is important. Whether the antibacterial polymer coatings are effective for prevention of IRIs or not needs clinical validation.

## Abbreviations

ACC: Antibiotic cement-coated
AFM: Atomic force microscopy
BCP: Bi-phasic calcium phosphates
CFU: Colony-forming units
CPC: Calcium phosphate cements
CWA: Cell wall-anchored
EPS: Extracellular polymeric substances
ETN: Expert Tibia Nail
FDA: American Food and Drug Administration
HA: Hydroxyapatite
IRIs: Implant related infections
LBL: Layer-by-layer
MBC: Minimal bactericidal concentration
MIC: Minimal inhibitory concentrations
MRSA: Methicillin resistant *Staphylococcus aureus*
NOAEL: No observable adverse effect level
PAA: Poly (acrylic acid)
PDLLA: Poly(D,L-lactic acid)
PEG: Polyethylene glycol
PEO: Polyethylene oxide
PIA: Polysaccharide intercellular adhesin
PLEX: Polymer-Lipid Encapsulation MatriX
PLLA: Poly(lactic acid)
PMMA: Polymethylmethacrylate
PSMs: Phenol-soluble modulins
PVA: Poly (vinyl alcohol)
RGD: Arginine-glycine-aspartic acid
SDS: Sodiumdodecylsulfate
SEM: Scanning electron microscope
β-TCP: Beta-tricalciumphosphate
